# Toward Real Scenery: A Lightweight Tomato Growth Inspection Algorithm for Leaf Disease Detection and Fruit Counting

**DOI:** 10.34133/plantphenomics.0174

**Published:** 2024-04-15

**Authors:** Rui Kang, Jiaxin Huang, Xuehai Zhou, Ni Ren, Shangpeng Sun

**Affiliations:** ^1^ Institute of Agricultural Information, Jiangsu Academy of Agricultural Sciences, Nanjing 210044, China.; ^2^ Bioresource Engineering Department, McGill University, Montreal, QC H9X 3V9, Canada.

## Abstract

The deployment of intelligent surveillance systems to monitor tomato plant growth poses substantial challenges due to the dynamic nature of disease patterns and the complexity of environmental conditions such as background and lighting. In this study, an integrated cascade framework that synergizes detectors and trackers was introduced for the simultaneous identification of tomato leaf diseases and fruit counting. We applied an autonomous robot with smartphone camera to collect images for leaf disease and fruits in greenhouses. Further, we improved the deep learning network YOLO-TGI by incorporating Ghost and CBAM modules, which was trained and tested in conjunction with premier lightweight detection models like YOLOX and NanoDet in evaluating leaf health conditions. For the cascading with various base detectors, we integrated state-of-the-art trackers such as Byte-Track, Motpy, and FairMot to enable fruit counting in video streams. Experimental results indicated that the combination of YOLO-TGI and Byte-Track achieved the most robust performance. Particularly, YOLO-TGI-N emerged as the model with the least computational demands, registering the lowest FLOPs at 2.05 G and checkpoint weights at 3.7 M, while still maintaining a mAP of 0.72 for leaf disease detection. Regarding the fruit counting, the combination of YOLO-TGI-S and Byte-Track achieved the best *R*^2^ of 0.93 and the lowest RMSE of 9.17, boasting an inference speed that doubles that of the YOLOX series, and is 2.5 times faster than the NanoDet series. The developed network framework is a potential solution for researchers facilitating the deployment of similar surveillance models for a broad spectrum of fruit and vegetable crops.

## Introduction

Tomatoes abound in a rich of essential nutrients, positioning them as one of the most popular fruits worldwide [1]. With the increasing adoption of modern breeding techniques and advanced automated fertilization technologies in greenhouses, there has been a notable upsurge in both tomato yield and quality [2]. However, due to the intensive planting practices employed, several contagious diseases have the potential to swiftly propagate among tomato plants, posing a substantial threat to sustainable production [3]. An elucidative report underscores that the direct production losses attributed to various diseases could exceed approximately 30% [4]. Furthermore, diseases affecting the foliage may spread onto the fruit surface, resulting in fruit deformities and thereby exerting a deleterious impact on economic value.

In response to these challenges, several artificial intelligence and machine vision-based approaches have been deployed to address the detection and analysis of tomato plant diseases [5]. Since early symptoms of certain plant diseases manifest on leaves, the utilization of diverse algorithms for leaf-based assessment and identification proves to be a more efficient tool of inferring the plant’s health status [6]. A dataset known as Plant Village, comprising dozens of tomato diseases, has been established and used to evaluate various benchmark convolutional neural networks (CNNs) [7]. For the characterization of leaf diseases, plenty of classification algorithms such as LeNet [8] and ResNet [9] have been implemented and achieved outstanding scores in accuracy. However, the practical intricacies of tomato cultivation in the real world introduce a heightened level of complexity. Dynamic fluctuations stemming from factors such as lighting conditions, plant density, and the complicated leaf growth have been demonstrated to pose challenges to the effectiveness of such algorithms in real-world applications [10]. To validate these algorithms with the authentic conditions of tomato growth, Singh *et al.* [11] embarked on the collection of leaf images from agricultural environments. This endeavor led to the creation of the PlantDoc dataset, which includes a wide variety of 13 different plant species. Based on this dataset, several state-of-art detection algorithms were tested and attained a modest score of 0.37 (mAP).

In real-world scenarios involving leaf analysis, detection algorithms demonstrate more complexity than disease classification algorithms. These networks play a multifunctional role that transcends simple object categorization, encompassing the crucial task of providing spatial location information of the targets [12]. Presently, the detection networks can be broadly categorized into two primary categories: region-based two-stage convolutional neural networks (RCNNs) [13] and one-stage networks represented by the YOLO series [14]. Notably, the performances of YOLO series are primarily due to their lightweight architecture, making them exceptionally well suited for deployment in the applications. The YOLO networks typically comprise feature extraction backbones and specialized heads for diverse target tasks. By leveraging an end-to-end training paradigm, they effectively streamline parameter complexity and have the capability to concurrently predict both the position and category of target objects. Through refinements applied to the backbone, attention mechanisms, and loss functions, the YOLO series algorithms have demonstrated their capability to achieve precise detection of small disease regions on tomato leaves. For example, Wang refined the YOLOv3 network with MobileNetv2 [15], while Tang and collaborators introduced PLPNet [16], a solution module adept at mitigating soil-related interference. These research findings underscored the practicality of employing YOLO networks for tomato leaf detection. Nevertheless, they offer limited insight into the performance of detection algorithms under real-world conditions. The efficacy of visual models is contingent upon a complex interplay of factors, encompassing model architecture, fine-tuning of hyperparameters, and deployment strategies [17]. While disparities in model architecture are readily observable, critical technical intricacies associated with hyperparameter configuration and deployment strategies are often undisclosed, making hard for others to replicate results on customized datasets.

Within the realm of tomato production monitoring, the fast and accurate quantification of tomato yields stands as a pivotal yet unresolved challenge [18]. Conventional visual detection methods heavily rely on color variations to perform threshold-based tomato segmentation and subsequent counting. For instance, manually crafted features such as color-based thresholding methods were commonly employed to delineate tomato regions. These methodologies exhibit limitations, particularly in scenarios marked by fluctuating environmental lighting conditions, resulting in substantial omission errors [19]. Recent deep learning-based detection approaches, which capitalize on robust high-dimensional features, have been successfully performed in the counting task targeted at apples and tomatoes [20]. These methods excel in the segmentation and counting of individual fruits within single images. Their capabilities fall short when confronted with the task of aggregating yield statistics across the entire planting areas. In greenhouse environments, tomatoes are typically cultivated in rows, and the utilization of video scanning presents a straightforward and rapid approach for fruit data acquisition. Nevertheless, accurately counting fruits from dynamic video footage remains a major technical challenge. Inspired by the simple online and real-time tracking (SORT) technique succussed in the video processing, several researchers have conducted the application of the Kalman filtering algorithm for fruit counting tasks [2,3]. Nonetheless, owing to a multitude of factors including the capability of base models, leaf occlusion, and computational resource constraints, current algorithms are still unable to achieve superior performance in the task of counting tomatoes based on video stream method.

In the domain of leaf disease detection and fruit tracking and counting, we introduce an algorithm that could integrate both functionalities simultaneously in this study. Specifically, for the detection of tomato leaf diseases, we employ a fixed-point static method to acquire high-resolution leaf images, subsequently performing categorization using a detector. For tomato fruit detection and counting, a high-speed inspection approach, employing mounted cameras, is deployed to facilitate the mass scanning of tomato plants. Based on the shared feature extraction model, the improved SORT method will be employed to accomplish tracking and counting within each frame of the video. The key contributions are summarized as follows: (a) We built an RGB image dataset featuring tomato disease-infected leaves and fruits. All the images were captured within an authentic greenhouse environment. (b) A new type of YOLO series network named YOLO-TGI was introduced for tomato growth inspection, with the specific Ghost module and convolutional block attention module (CBAM) designed for addressing leaf occlusion during fruit detection. (c) Furthermore, a unified framework integrating detection and tracking algorithms was developed for the task of tomato fruit tracking and counting, as well as testing their practical applicability in real-world scenarios.

## Materials and Methods

### Field data collection and preprocessing

**Fig. 1.**
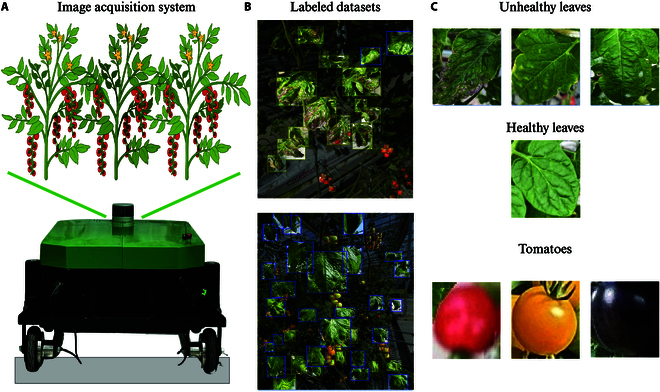
The image acquisition process in a tomato field. (A) Image acquisition system. An autonomous platform is utilized for capturing images and video scan of tomato plants. (B) Representative examples of labeled images. This dataset is annotated through the Roboflow platform. (C) Categories of the dataset. Instances of unhealthy leaves, healthy leaves, and tomato fruits are showcased within the dataset.

The images of tomato leaves and fruits were captured in the intelligent greenhouse facilities of Jiangsu Agricultural Science Academy in 2023. The tomato plants were nourished using a blend of cocopeat and an advanced nutrient fertilization system. An autonomous mobile platform was employed, outfitted with a commercial high-resolution smartphone camera (iPhone 12 Pro) mounted on a specialized bracket for data acquisition, capturing images at an original resolution of 3,024 × 4,032 pixels (Fig. 1A). Annotation of these images was facilitated using the Roboflow platform (Fig. 1B) [21]. The dataset (https://universe.roboflow.com/jaas/leaf-and-tomato) encompasses leaf diseases such as the bacteria spot disease, mosaic virus, late blight, and spider mold leaves. The detection targets within the dataset were categorized into unhealthy leaf, healthy leaf, and tomato fruit (Fig. 1C). Utilizing the ImgAug plugin [22], we augmented the annotated dataset via rotations, reflections, and translations, and subsequently resized the images to 640 × 640 pixels. Annotation structures were converted into both COCO and YOLO formats. The final dataset comprises 5,280 training images, 1,320 validation images, and 660 test images. To avoid the potential data leakage, the training, validation, and test datasets were stored in the different folders. Throughout the experimentation phase, the yaml file was utilized for data retrieval, guaranteeing the complete invisibility of test data during the model training process. Additionally, video stream of tomatoes was amassed for fruit counting tasks, with manual annotations serving as ground truth against algorithmic predictions. The detailed image acquisition works were summarized in the Supplementary Materials.

### Development of the detection and tracking pipeline

In our detection framework, we considered YOLO-v8, YOLOX [23], and NanoDet-Plus [24] as candidate detectors, while Byte-Track [25], Motpy [26], and FairMot [27] were implemented as candidate trackers. The primary role of the detection networks lies in identifying the leaf health status and the fruit bounding boxes, whereas the tracking networks facilitate the continuous detection by updating the fruit’s position within video sequences. Contemporary detection models frequently draw upon their specific intricate architectures to augment network performance. For instance, YOLOX introduced anchor-free design to replace the anchor-based part, which is the essential part of YOLO series, leveraging the efficiencies of feature proposal networks and focal loss algorithms to enhance feature detection. Similarly, YOLO-v8 adopted the anchor-free approach, introducing a method centered around a center-based paradigm. Conversely, NanoDet employs the ShuffleNet-v2 architecture as its backbone, striking an optimal balance between model speed and accuracy. To harmonize detection precision and processing speed, we instituted specific improvements to the backbone of YOLO-v8. Details were described as follows.

**Fig. 2.**
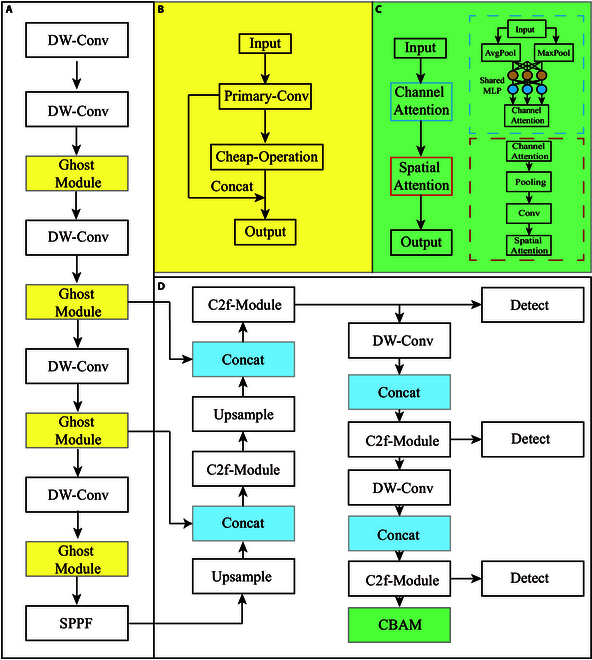
The architecture of YOLO-TGI network. (A) Backbone of YOLO-TGI. (B) Ghost modules. (C) CBAM modules. (D) Head of YOLO-TGI. The yellow blocks represent the utilization of Ghost modules for replacement, while the green blocks denote CBAM modules. Details of the internal computations for these newly added network modules are showcased in the top right corner. The blue blocks denote the features of Ghost modules that will be concatenated for the next processing module. The aim of the three detecting blocks is to extract features of various scales to enhance the model’s robustness.

1. Replacing ordinary convolutional layers with Ghost modules. Drawing inspiration from GhostNet [28], we incorporated the deep Ghost module (yellow block in Fig. 2A) into the YOLO-v8 architecture to reduce network size and computational overhead. As shown in Fig. 2B, the Ghost module (the yellow block) initiates with 1 × 1 primary convolutions (Primary-Conv) to compress feature channels and subsequently employs a cheap operation for layered convolutions, yielding an increased number of feature maps. The cheap operation in Ghost module primarily relies on linear transformations, enabling the production of additional feature maps (also named as “ghost features”) through elementary computations. This improvement of cheap computation allows the Ghost module to markedly reduce both the computational load and the number of parameters in the model. Leveraging the Ghost module to generate an equivalent number of feature maps as a conventional convolutional layer, we seamlessly removed the convolutional layers and integrated the depth-wise separable convolution (DW-Conv) into the existing neural network framework (Fig. 2A), thereby diminishing computational burdens [29]. Furthermore, the classic structures of C2f module and spatial pyramid pooling fast (SPPF) module were kept in the establishment of YOLO-TGI network. The C2f module is kept in enhancing gradient flow and feature utilization efficiency during the network backpropagation, while the SPPF module is employed for the extraction and integration of features across different scales.

2. Addressing leaf occlusion issues. Traditional detection algorithms grapple with the challenge of leaves obstructing fruits, primarily attributed to the sensitivity of nonmaximum suppression (NMS) thresholds, leading to missed detections. Our devised CBAM amplifies the network’s capacity to discern fruits concealed by leaves. CBAM module primarily operates on two mechanisms: spatial attention and channel attention, which allow it to adaptively focus on the main parts of an image, thereby enhancing the model’s representational capability [30]. As shown in Fig. 2C (the green block), the feature map *F* with dimensions *H × W × C* undergoes separate processes of global max pooling (MaxPool) and global average pooling (AvgPool) along its width and height, respectively, yielding two feature maps of dimension 1 × 1 × C. These condensed feature maps are then each fed into a shared two-layer neural network, which is also named as shared multilayer perceptron (MLP). After MLP processing, the features are combined via element-wise addition, followed by a sigmoid activation to yield the channel attention feature (Channel Attention). The final step involves an element-wise multiplication with the original input feature map *F*, furnishing the requisite input features for the Spatial attention module. The integration of the CBAM module endows the YOLO-TGI network with the capability to autonomously focus on and amplify pertinent features. This attention-driven approach refines the network’s ability to discern and prioritize crucial aspects of the target, even in complex visual environments with obstructions. Specifically, the adaptive mechanism enables the YOLO-TGI to highlight key characteristics of partially obscured objects, thereby effectively addressing the issue of fruit detection in scenarios where fruits are partially concealed by leaves.

**Fig. 3.**
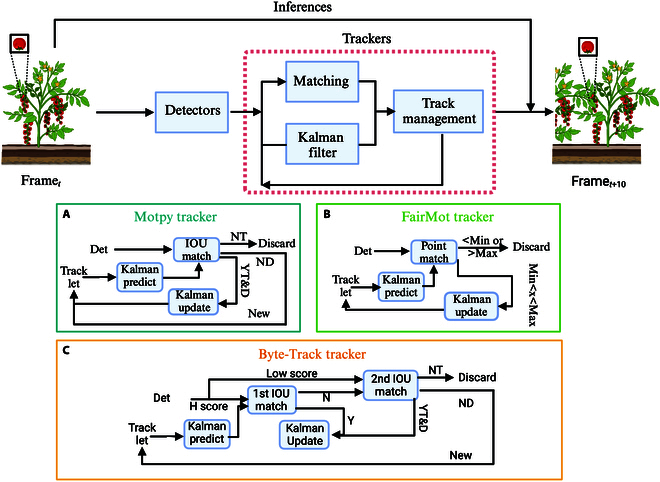
The implementation mechanism of trackers. Internal computational logic of the (A) Motpy tracker, (B) the FairMot tracker, and (C) the Byte-Track.

Current outperforming multi-object tracking (MOT) methods applied tracking by detection paradigm, which consisted of two pivotal functionalities: object detection and re-identification (Re-ID). The base detector was employed to precisely discern the location of targets within each individual frame, whereas the Re-ID process establishes intricate associations between current targets and their antecedent counterparts in prior frames. As shown in Fig. 3, the tracking modules incorporated in our work were formatted in modular design, predominantly comprising the Hungarian matching algorithm, Kalman filters, and track management for state updates [31]. The Hungarian matching algorithm meticulously resolves issues of cascaded matching across consecutive frames, while Kalman filters proffer predictive insights into the anticipated positions and velocities of the targets. Within this paradigm, both Motpy and Byte-Track networks utilize intersection over union (IOU) between detected and Kalman-predicted bounding boxes as a cost function, ensuring the seamless continuity of targets across frames. In the case of Motpy shown in Fig. 3A, successful matches culminate in the update of the extant track IDs. Conversely, failed matches necessitate the instantiation of a new track ID, followed by its initialization within the Kalman filter module. FairMot, which is shown in Fig. 3B, meticulously tracks changes in the trajectory of point positions, employing Kalman filters to predict the future locales of each point, and leveraging distance functions, such as the Euclidean distance, for alignment judgments. An inertia counter quantifies the frequency of matches for each point position on the detected object, with pre-established upper and lower thresholds dictating the decision to maintain or dissolve prolonged associations with the tracking entity. Byte-Track shown in Fig. 3C introduces a suite of enhancements, primarily through the categorization of detection boxes into groups of high and low confidence. High-confidence detection boxes are seamlessly integrated into the trajectories, while the strategic association between low-confidence detection boxes and unmatched tracking entities mitigates the challenges posed by missed detections.

To enhance the accessibility and streamline the utilization of both the detector and tracker, we have meticulously exported all varieties of trained detector models into the open neural network exchange (ONNX) format [32]. By applying ONNX format, various trained models from different frameworks or tools can be easily moved for further purposes. Currently, we have built the pertinent initiation files for each tracker to realize a seamless integration. The fine-tuning of various parameters is adeptly handled through configuration files (in json format), allowing for nimble modifications. The complexity of the YOLOX, NanoDet, and YOLO-TGI detectors can also be adjusted through config files (currently support nano N, small S, and medium M). Detailed insights into the dataset and code can be reached from the repository (https://github.com/RuiKangnj/TGI/tree/main).

### Evaluation metrics

To evaluate the performances of our detection models, metrics such as the floating point operations per second (FLOPs), mAP, parameters (M), and the inference speed (ms) were employed [33]. As shown in Eq. 1, *p* denotes the precision value and *r* denotes the recall value. For multi-class classification tasks, the AP of each class and then take the average calculation to obtain the final mAP (Eq. 2). For base detectors, we tested their respective performances using the image dataset. However, for the tracking models, we used videos for fruit counting evaluation. These videos were taken by scanning the tomato planting site with a smartphone, containing a varying number of tomato fruits. The counting results of the test videos were compared to manual counting results by employing the coefficient of determination (*R*^2^; following Eq. 3) and root mean square error (RMSE; following Eq. 4). The proposed detection models were trained on a server running the Ubuntu operating system utilizing the A100 graphics processing unit (GPU, NVIDIA, 40 GB memory), PyTorch 1.12, and CUDA 10.2. The training process incorporated an adaptive adjustment of learning rates using an stochastic gradient descent (SGD) strategy complemented by momentum, and featured functionalities for automatic weight saving and early stopping. The specific details were also provided in the code. We obtained the models’ FLOPs and Params through relevant interfaces, while the mAP was calculated based on the independent test set. The inference speed of the models is jointly determined by the network’s complexity, hardware, and the resolution of the processing images. As such, we utilized a Mac notebook with M1 CPU, 16 GB memory, and PyTorch 1.13 to conduct the tracking assessments.

$$\text{AP}=\int_{0}^{1}p\left(r\right)\mathrm{dr}$$ (1)

$$\text{mAP}=\frac{1}{N}\sum_{i=1}^{N}{\mathrm{AP}}_{i}$$ (2)

$$R^{2}=1-\frac{\sum {\left(y_{i}-{\hat{y}}_{i}\right)}^{2}}{\sum {\left(y_{i}-\bar{y}\right)}^{2}}$$ (3)

$$\text{RMSE}=\sqrt{\frac{1}{n}\sum_{i=1}^{n}{\left(y_{i}-{\hat{y}}_{i}\right)}^{2}}$$ (4)

## Results and Discussion

### Performance of base detectors

**Fig. 4.**
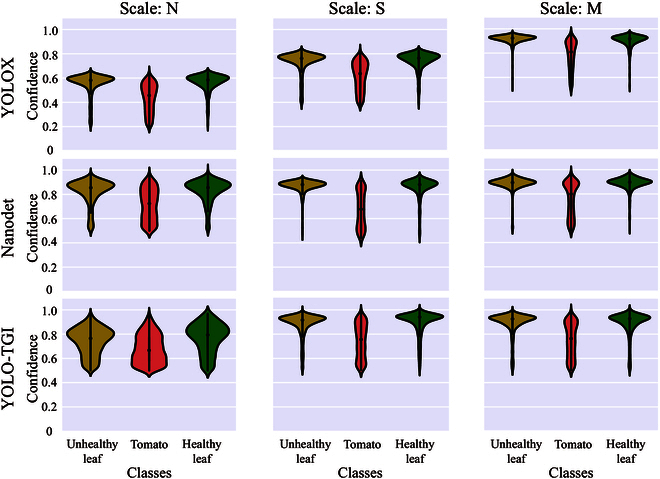
The confidence distribution trends of various base models across multiple classes at scales N, S, and M. In this context, a model with better performance should exhibit a narrower “violin” section in the plot, and the denser portion of it should be located in the higher value area.

The evaluation of detection accuracy for the YOLOX, NanoDet, and YOLO-TGI algorithms was conducted on the test dataset. Confidence distributions for categories of unhealthy leaf, healthy leaf, and tomato were represented through violin plots (Fig. 4). It was observed that an augmentation in the complexity of the network tends to be associated with an enhancement in detection accuracy across the detectors examined. Particularly, YOLOX-M was seen to achieve the optimal confidence distribution, with a predominant concentration of leaf detection confidence scores situated around 0.9, and those for tomato detections lying in the range of 0.6 to 0.9. In contrast, YOLOX-N was found to exhibit the most inferior confidence distribution, characterized by a variability of scores from 0.2 to 0.7. This suboptimal performance was likely attributable to its employment of a more lightweight network structure, which was speculated to lead to a drastic reduction in detection capabilities. NanoDet and YOLO-TGI were observed to display nearly analogous trends in confidence distribution, with both detectors producing confidence scores above 0.4. It merits emphasis that a greater robustness in detection outcomes for both healthy and unhealthy leaves was demonstrated by NanoDet and YOLO-TGI, compared to the tomato detections, which presented a bimodal distribution of confidence scores. For some base detection models, the values at the top of the violin plots were observed to slightly exceed 1.0. This is due to the confidence prediction values of these models being tightly clustered and close to 1 when applying kernel functions for data visualization. The confidence distribution in the violin plots is beneficial for us to visually understand the performance of various detectors on different classes of targets, and it also assists in selecting more suitable threshold values for actual classification.

To further elucidate the performance characteristics of the detectors, a comprehensive summary of the data has been collated in Table 1. It was noted that YOLOX, within its N and S scale network architectures, operates at a resolution of 416 × 416, while NanoDet utilizes a resolution of 320 × 320 at the S scale, with the remaining detectors functioning at a resolution of 640 × 640. Generally, higher-resolution inputs are associated with the capture of more detailed image information, which typically translates to increased detection precision; however, this is often at the expense of reduced inference speed and a reliance on more substantial computational resources. Each detection model demonstrated distinct advantages within varying attributes. For instance, the YOLOX model achieved the fastest inference speed at 32.35 ms and the highest mAP of 0.85. NanoDet, on the other hand, boasted the smallest model size with parameters tallying to 1.16 M, whereas YOLO-TGI had the lowest number of FLOPs at 2.05 and checkpoint weights amounting to 3.7 M.

**Table 1.** The summary of inference results of various base detectors

Base detection models	Resolution	Backbone	FLOPs (G)	Param (M)	Checkpoint weights (M)	mAP	Inference speed (M1-CPU) (ms)
YOLOX-N	416 × 416	C2f	4.93	2.24	9.00	0.50	32.35
YOLOX-S	416 × 416	C2f	6.44	5.03	20.20	0.68	38.34
YOLOX-M	640 × 640	C2f	26.76	8.94	35.80	0.85	99.3
NanoDet-N	640 × 640	ShuffleNet-v2-1.0x	18.40	1.16	5.50	0.78	166.08
NanoDet-S	320 × 320	ShuffleNet-v2-1.5x	8.95	2.43	10.60	0.81	72.3
NanoDet-M	640 × 640	ShuffleNet-v2-1.5x	35.8	2.44	10.60	0.83	245.40
YOLO-TGI-N	640 × 640	GhostNet	2.05	1.59	3.70	0.72	54.20
YOLO-TGI-S	640 × 640	GhostNet	6.08	5.43	21.90	0.83	88.20
YOLO-TGI-M	640 × 640	GhostNet	12.3	11.6	46.80	0.83	143.00

**Fig. 5.**
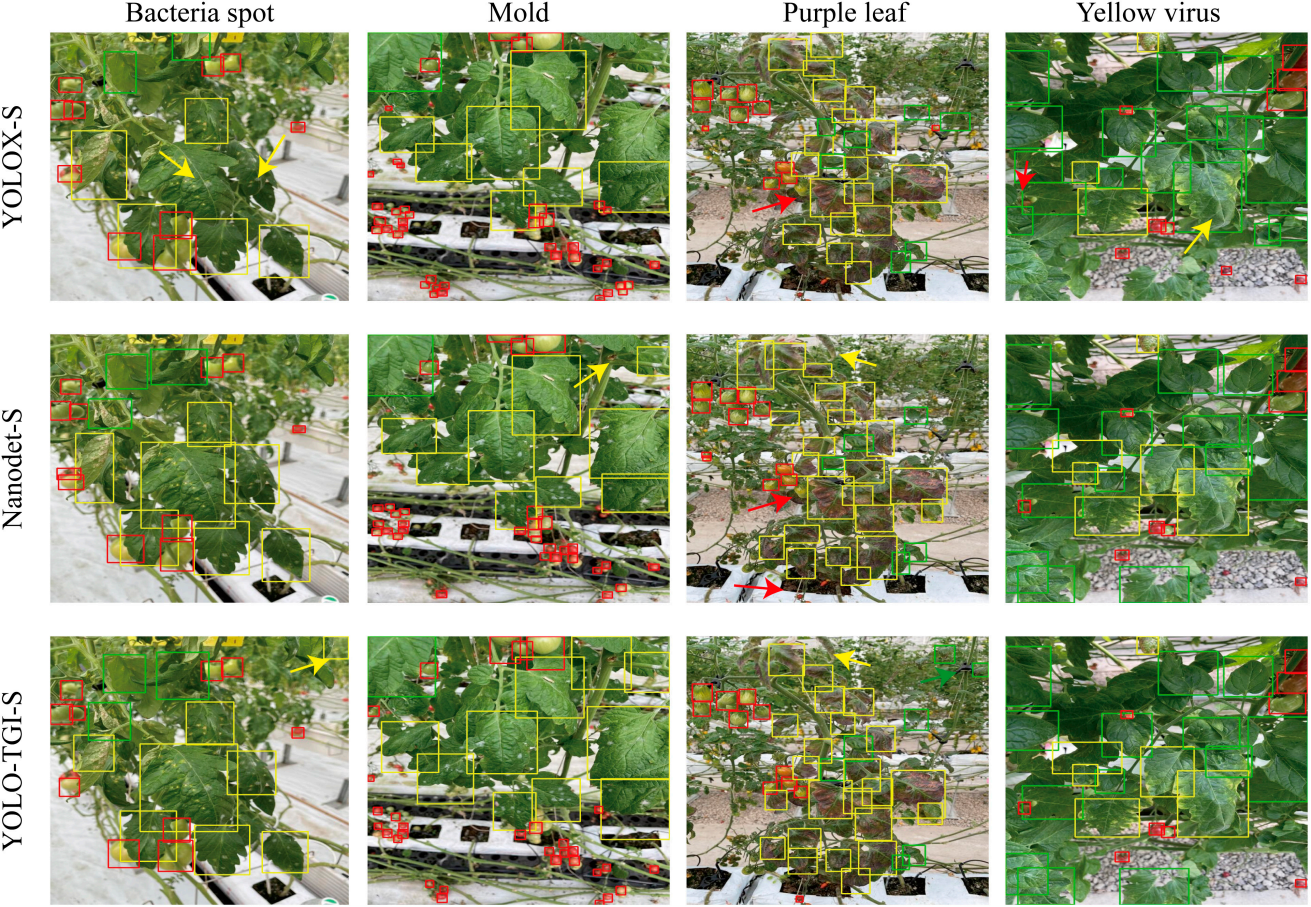
Inference results of various base detection models in leaf disease and tomato fruit detection tasks. The yellow rectangular boxes indicated unhealthy leaves, green rectangular boxes represented healthy leaves, and red rectangular boxes denoted the tomato fruit area. The performance of the network was characterized by rectangular boxes accurately encompassing the specific target pixel areas.

Furthermore, the efficacy of different detectors was tested on the task of identifying four categories of leaf diseases. Their specific inference results are shown in Fig. 5. The colors red, green, and yellow denote tomatoes, healthy leaves, and unhealthy leaves, respectively. In the case of bacterial spot detection, both YOLOX-S and NanoDet-S models exhibited omission errors, while the YOLO-TGI-S model demonstrated heightened sensitivity to small regions, all of which were indicated by yellow arrows in the imagery. Notably, the YOLOX-S model was prone to marked misidentification during the detection of leaves with yellow virus, mistakenly classifying unhealthy leaves as healthy (labeled in yellow arrows). The NanoDet-S model tended to overlook tomatoes, suggesting a lack of sensitivity to tomatoes obscured by foliage (labeled in red arrows). The YOLO-TGI-S model, augmented with the CBAM module, showed excessive sensitivity. For instance, leaves designated as background in the case of purple leaf disease were erroneously assessed. After further inspection of the incorrectly detected cases (labeled in arrows in different colors), most false detections were subjected to complexities such as blurring, partial concealment of lesions, and stem infections in the field, leading to indeterminate or ambiguous target regions. These instances were more prevalent among healthy leaves, where detectors failed to draw rectangles on many small healthy leaves, since these were often trained as background in the original dataset. However, the detection of unhealthy leaves was notably precise, owing to the abundant annotations for such samples in the dataset. Hence, the imbalance in sample quantity had a slight potential impact on the results, an effect that was challenging to mitigate in real-world scenarios due to the prevalence of fragmented leaves throughout the growth of tomatoes.

### Fruit counting results of the tomato tracker scanning

**Fig. 6.**
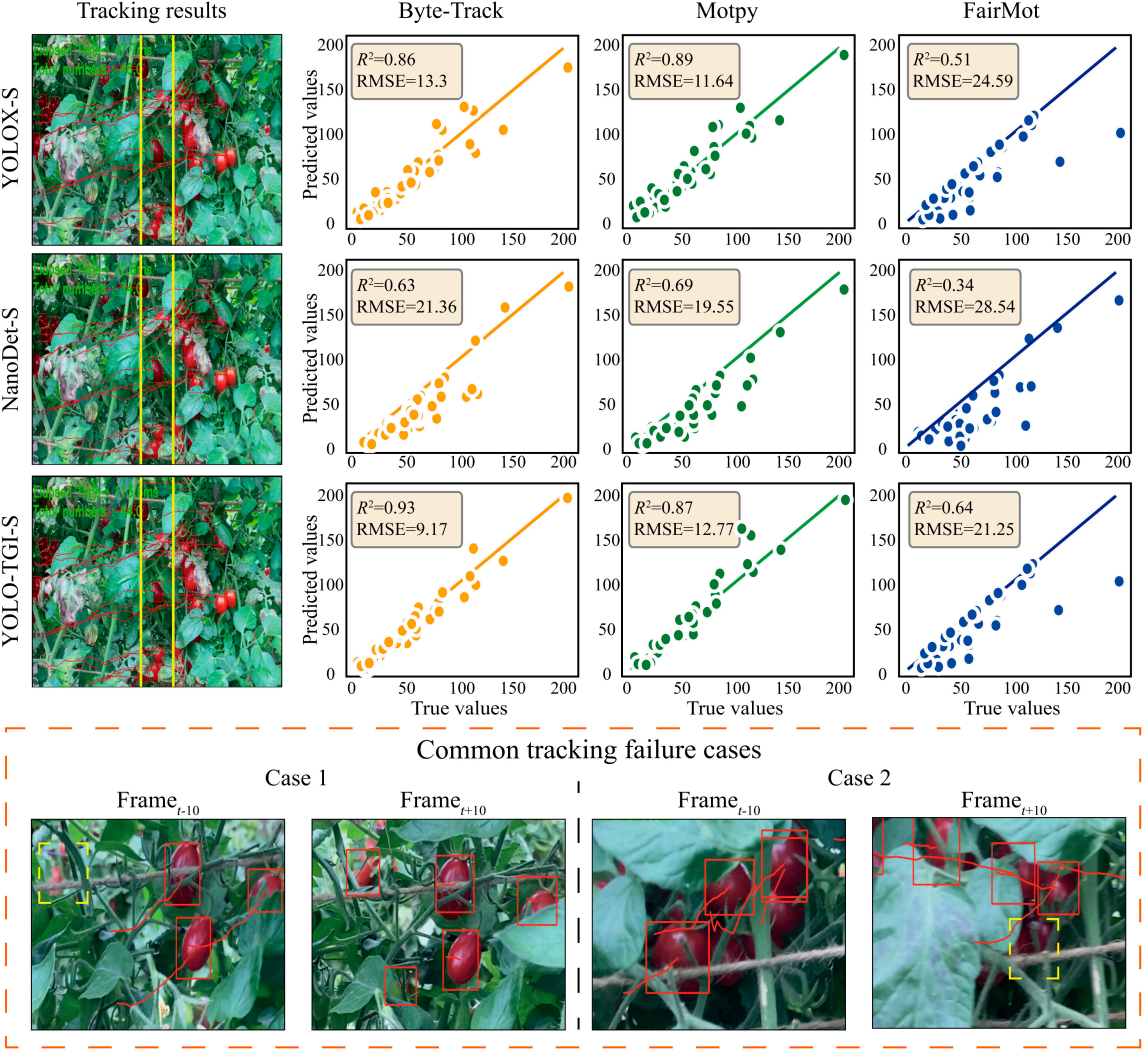
Scattering plot of predictions and ground truth for various detector-trackers and instances of tracking failures. A good tracker should exhibit the highest *R*^2^ value and the lowest RMSE. The tracking failure cases were primarily characterized by the inability to continuously track a specific target during video frame transitions.

To track the regions of tomato fruits within video streams, we adopted a cascading approach that integrates detectors with trackers. Representative tracking scenarios are depicted in Fig. 6, where the regions of tomatoes were delineated with red bounding boxes, and their motion trajectories were plotted based on the coordinates of the box centers. Video demonstrations showcasing the performance of three categories of detectors on tomato detection can be reached via the link of the code repository. While the tracking algorithms were capable of continuous tracking for most of tomato regions, instances of missed and erroneous detections were still evident. The detection images on the left side of Fig. 6 illustrated some of these occurrences. For tomatoes unobscured by foliage, all types of detection trackers were successful in recognizing and following the tomato regions. However, in some results, leaves afflicted with disease such as purple leaves of yellow bacteria exhibited textural characteristics similar to those of unripe tomatoes (fruits with yellow or black color). This issue often exacerbated in video streams of low resolution. Another interesting observation was the inadvertent enhancement of recognizability by specular reflections on the fruit surfaces, which presented a distinguishable gloss signal not replicable by either healthy or diseased leaves.

To illustrate the impact of scale on detection, we modeled the total count predictions generated by the tracking algorithms against the ground truth, with the regression comparison results of Byte-Track, Motpy, and FairMot displayed on the right side of Fig. 6. The combination of YOLO-TGI-S with Byte-Track manifested the highest coefficient of determination *R*^2^ at 0.93 and the lowest RMSE at 9.17, while NanoDet-S coupled with FairMot yielded the lowest *R*^2^ at 0.34 and the highest RMSE at 28.54. Within the same tracking framework, both YOLO-TGI-S and YOLOX-S markedly outperformed the NanoDet-S model. In Fig. 6, scatterplot trends revealed minimal variance among models for test videos with a lower total count of tomatoes. However, as the tomato count numbers increased, a substantial deviation between predicted values and ground truth emerged, indicating the potential for compounded errors in tracking tasks. Besides, interference from the background and foliage was identified as the primary contributor to these discrepancies. As depicted in Fig. 6 (case 1), in the Frame_*t*−10_ image, some tomatoes were occluded by leaves and therefore not classified by the detector (labeled in yellow dashed bounding box). Yet, at Frame_*t*+10_, as the camera moved, these regions were detected and marked, leading to an inflated count. Conversely, certain tomato regions fully visible in Frame_*t*−10_ became partially or completely obscured due to camera movement and changing angles of capture, resulting in a reduced count (shown in case 2 in yellow dashed bounding box). To mitigate such ID-switching scenarios, we incorporated an ID-filtering strategy based on virtual boundary ranges, indicated by the yellow lines in the imagery, which enforces counting only when tomato IDs enter this designated area.

Table 2 reports the performance outcomes achieved through the integration of various classifiers with trackers. The combination of YOLO-TGI-S and Byte-Track was reported to have attained the optimal *R*^2^ value of 0.93 and the minimum RMSE of 9.17, while the YOLOX-N and Motpy ensemble exhibited the most rapid inference time, recorded at 49.8 ms. In terms of tracker performance, FairMot was observed to be suboptimal, with the YOLOX-N–FairMot pairing yielding the lowest *R*^2^ of 0.32 and an RMSE of 29.13, and the YOLO-TGI-M–FairMot combination obtaining an *R*^2^ of 0.69 and an RMSE of 19.48. The capability of FairMot to support the task of tomato detection and tracking was suggested to be inadequate, with Byte-Track being better suited for integration with YOLO-TGI, and Motpy more compatible with YOLOX-N. The inference time served as a critical metric for objective detection models, signifying the alacrity with which detections were made, a factor of quintessential importance for real-time applications. It was noted that the N and M scale models of YOLOX achieved the fastest and slowest detection speeds at 49.8 and 308.1 ms, respectively, indicating that network architecture selection must be meticulously considered during application deployment. At the S and M scales, the YOLO-TGI–Byte-Track combination was found to have a substantial advantage in terms of operational speed while preserving high accuracy, being nearly twice as fast as YOLOX and about 2.5 times faster than NanoDet. Paradoxically, an increase in network scale from N to M was accompanied by a decrease in the precision of YOLO-TGI detections. Further investigation attributed this situation to the incorporation of the CBAM network, which resulted in a greater number of fragmented regions being incorrectly identified as tomato fruit regions, which were originated from the background and not annotated in the manual labeling process.

**Table 2.** The inference results on real video streams of tomato fruits of different detector-tracker combinations

	Tracker	*R* ^2^	RMSE	Inference speed (ms)
YOLOX-N	Byte-Track	0.84	13.88	56.9
	Motpy	0.77	16.91	49.8
	FairMot	0.32	29.13	61.3
YOLOX-S	Byte-Track	0.86	13.3	192.6
	Motpy	0.89	11.64	209.6
	FairMot	0.51	24.59	201.3
YOLOX-M	Byte-Track	0.84	13.96	283.6
	Motpy	0.90	11.23	303.8
	FairMot	0.49	25.26	308.1
NanoDet-N	Byte-Track	0.78	16.58	267.9
	Motpy	0.86	13.87	270.6
	FairMot	0.57	23.03	276.4
NanoDet-S	Byte-Track	0.63	21.36	249.2
	Motpy	0.69	19.55	248.6
	FairMot	0.34	28.54	249.6
NanoDet-M	Byte-Track	0.89	11.79	269.0
	Motpy	0.85	13.82	268.6
	FairMot	0.65	20.82	282.4
YOLO-TGI-N	Byte-Track	0.90	11.32	115.7
	Motpy	0.88	12.38	103.0
	FairMot	0.63	21.55	110.2
YOLO-TGI-S	Byte-Track	0.93	9.17	118.2
	Motpy	0.87	12.77	107.0
	FairMot	0.64	21.25	111.8
YOLO-TGI-M	Byte-Track	0.86	13.29	121.0
	Motpy	0.84	14.06	106.7
	FairMot	0.69	19.48	112.9

In conclusion, the highest detection accuracy was manifested by the YOLO-TGI-S–Byte-Track configuration, with *R*^2^ and RMSE values reported at 0.93 and 9.17, respectively.

## Discussion

The monitoring of plant growth conditions in production environments is a challenging endeavor, compounded by the variable nature of disease patterns, intricate background contexts, and complex lighting conditions. This study unveils a novel resource: a comprehensive, annotated database of tomato leaves and fruits designed for real-world scenarios. The database captures the dynamic postures of growing tomato plants, encompassing a variety of diseases, and features diverse backgrounds that facilitate the training of deep learning algorithms tailored for in-field application. Presently, there are only two extensive annotated datasets for tomato leaf images: Leaf Village and Plant Doctor. The dataset we have constructed serves a distinct purpose from these two. Its primary advantage lies in its focus on the health status of tomato leaves throughout the growth cycle and the potential fruit yield. Leaf Village predominantly consists of images of individual leaves from various plant species. Numerous studies have pointed out that the homogeneous backgrounds in these data do not favor the training of robust models [34]. While Plant Doctor does offer some data from real-life settings, the lack of a large-scale, proprietary cultivation greenhouse means that many of its images are randomly sourced from the internet, failing to reflect authentic contexts, thereby limiting its utility in constructing detection models for field applications. Our detection results also demonstrated the potential value of this large and diverse dataset, particularly in the training of robust detection networks. The veracity of the dataset in depicting real agricultural settings enhances its relevance and applicability for developing algorithms aimed at real-time, in-field monitoring and disease detection in tomato plants.

**Fig. 7.**
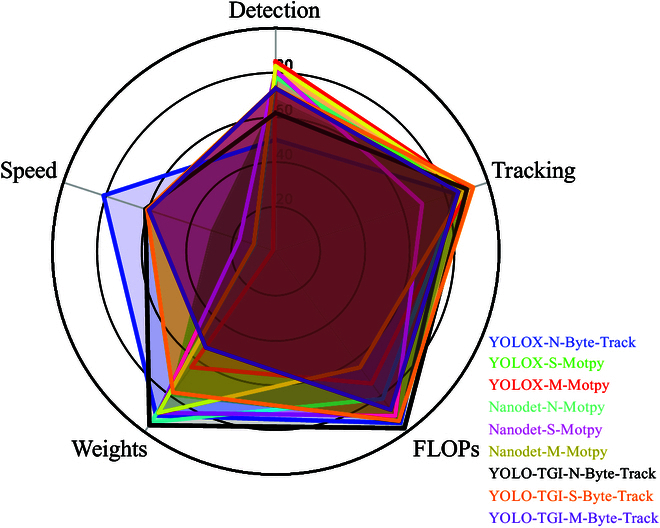
Radar chart illustrating the comprehensive performance of various detector-trackers. In a radar chart, the central point represents the lowest value of the attributes, while the edges signify the highest values.

In the domain of deploying machine learning models in real-world scenarios, the increasing complexity of network architectures presents substantial obstacles for implementation on farms. To evaluate the adaptability of various detection and tracking models during deployment comprehensively, we have synthesized a full analysis in Fig. 7. In this radar chart, each vertex represents a performance metric, and nine different colors denote distinct detection and tracking combinations that have achieved their respective optimal performances. We report on five metrics: detection score, tracking score, FLOP score, checkpoint weights score, and speed. The ideal network would occupy the entire pentagon, signifying optimal detection accuracy, tracking precision, and speed, with minimal checkpoint weights and FLOPs. The YOLOX-N–Byte-Track combination boasts the fastest detection speed but falls short in detection accuracy. NanoDet, designed specifically for mobile devices, is resource-efficient but lags in tracking precision and is among the slower networks. The YOLO-TGI series, incorporating CBAM for attentional mechanisms, leads in tracking scores, while its use of Ghost modules reduces the network’s parameter count, placing it at a moderate level in terms of checkpoint weights and FLOPs. Overall, the YOLO-TGI-S–Byte-Track network scores highly on most vertices and maintains an average score in calibration. Therefore, it emerges as a formidable contender for applications requiring high-speed and high-accuracy operations.

## Conclusion

Monitoring diseases and counting fruits are pivotal tasks in the cultivation of tomatoes, traditionally relying on labor-intensive manual visual inspections. Our research introduces a novel paradigm with the development of a lightweight detection and tracking model that offers a potential solution to these challenges. Utilizing mobile field-operating devices allows for the rapid acquisition of extensive data on tomato growth processes. The proposed YOLO-TGI series detection and tracking methodology facilitates prompt diagnosis of infections and quantitative yield estimation, providing invaluable tools for farmers and greenhouse managers. Throughout our research, we incorporated the CBAM module to address issues such as leaf occlusion and motion blur, while generally adopting Ghost modules to reduce the model’s parameters, thus achieving a more lightweight design. We have re-engineered the interfaces of various detection and tracking models to allow for the seamless integration of the most advanced networks within a unified framework. This integration paves the way for selecting optimal models tailored to the specific requirements of tomato growth monitoring tasks. Based on multiple technical enhancements to lightweight detection and tracking networks, continuous and quantitative monitoring of the growth state of tomatoes in cultivation fields were enabled. Notably, with retraining, this model has the potential to be extended to a broad spectrum of similar monitoring tasks for widely cultivated fruits and vegetables such as apples, oranges, grapes, and strawberries, underscoring its versatility and broad applicability.

## Data Availability

Dataset and code can be reached at https://github.com/RuiKangnj/TGI/tree/main.
